# Adenovector GAD65 gene delivery into the rat trigeminal ganglion produces orofacial analgesia

**DOI:** 10.1186/1744-8069-5-42

**Published:** 2009-08-05

**Authors:** Jean-Philippe Vit, Peter T Ohara, Christopher Sundberg, Blanca Rubi, Pierre Maechler, Chunyan Liu, Mariana Puntel, Pedro Lowenstein, Maria Castro, Luc Jasmin

**Affiliations:** 1 Department of Anatomy, University of California San Francisco, 513 Parnassus Avenue, San Francisco, CA 94143, USA; 2 Department of Cell Physiology and Metabolism, Faculty of Medicine, University of Geneva, Rue Michel-Servet 1, 1211 Geneva 4, Switzerland; 3 Gene Therapeutics Research Institute, Cedars-Sinai Medical Center, 8700 Beverly Boulevard, Los Angeles, CA 90048, USA; 4 Los Angeles Neurosurgical Institute, 8670 Wilshire Blvd, 201, Beverly Hills, CA 90211, USA

## Abstract

**Background:**

Our goal is to use gene therapy to alleviate pain by targeting glial cells. In an animal model of facial pain we tested the effect of transfecting the glutamic acid decarboxylase (GAD) gene into satellite glial cells (SGCs) of the trigeminal ganglion by using a serotype 5 adenovector with high tropisms for glial cells. We postulated that GABA produced from the expression of GAD would reduce pain behavior by acting on GABA receptors on neurons within the ganglion.

**Results:**

Injection of adenoviral vectors (AdGAD65) directly into the trigeminal ganglion leads to sustained expression of the GAD65 isoform over the 4 weeks observation period. Immunohistochemical analysis showed that adenovirus-mediated GAD65 expression and GABA synthesis were mainly in SGCs. GABAA and GABAB receptors were both seen in sensory neurons, yet only GABAA receptors decorated the neuronal surface. GABA receptors were not found on SGCs. Six days after injection of AdGAD65 into the trigeminal ganglion, there was a statistically significant decrease of pain behavior in the orofacial formalin test, a model of inflammatory pain. Rats injected with control virus (AdGFP or AdLacZ) had no reduction in their pain behavior. AdGAD65-dependent analgesia was blocked by bicuculline, a selective GABAA receptor antagonist, but not by CGP46381, a selective GABAB receptor antagonist.

**Conclusion:**

Transfection of glial cells in the trigeminal ganglion with the GAD gene blocks pain behavior by acting on GABAA receptors on neuronal perikarya.

## Background

Pain sensation most commonly results from the activation of peripheral branches of primary sensory neurons, the perikarya of which are located in either dorsal root ganglia (DRG) for body sensation or the trigeminal ganglia for sensation from the face. The central branches of sensory neurons in DRG terminate in the dorsal horn of the spinal cord and those of the trigeminal ganglion in the brainstem trigeminal nucleus. Injury to tissue or peripheral nerve induces central nervous system sensitization, facilitating pain processing responsible for allodynia and hyperalgesia [1,2]. A number of studies have shown that reducing the activity of primary afferents is often sufficient to alleviate peripherally generated pain conditions.

One approach to reducing neuronal activity is through the use of the inhibitory transmitter gamma-aminobutyric acid (GABA). Although there is an abundant literature showing the antinociceptive efficacy of GABA-acting drugs, most reports have been related to GABA manipulation in the central nervous system (CNS). Recently, however, Naik and colleagues [3] have shown that the application of GABA agonists to DRG led to a reduction of pain behavior in a model of sciatic nerve crush injury. The effect is theoretically not unexpected as there is evidence that both GABAA and GABAB receptors are expressed by primary sensory neurons in the trigeminal ganglia and DRG [4,5]. While it is likely that these receptors are principally exported to central terminals, some evidence suggest that they are also functional at the cell body within the ganglia [3,4]. On the basis that GABA receptors are present on neuronal perikarya in the ganglion, increasing GABA in the ganglia should reduce neuronal excitability and in pain conditions and potentially result in antinociception.

A recent strategy to induce GABA production has been to use viral vectors to introduce the synthetic enzyme for GABA, glutamic acid decarboxylase (GAD), into primary sensory neurons by inoculating the virus into subcutaneous tissue, to obtain retrograde transport to the sensory neuron bodies. The general advantage of this method is that the effects of gene based therapies are long lived without repeated dosing and are targeted to the affected area thus avoiding systemic effects. Peripheral inoculation of herpes simplex virus (HSV), leads to antinociception in a model of central neuropathic pain from spinal cord injury [6] as well as in a model of peripheral neuropathic pain after spinal nerve ligation [7]. More recently, the transfer of GAD67 to DRG neurons by peripheral inoculation of a novel human foamy virus (HFV) was shown to reduce nociceptive responses associated with spinal cord hemisection [6,8]. In these experiments increased expression of GAD67 mRNA was demonstrated in DRG and an increase in extracellular GABA was found in the spinal cord [8], suggesting that most of the analgesic effect resulted from GABA expression in sensory neurons followed by transport and release from terminals in the CNS.

In the present study, we wished to target glial cells to induce the production of GABA in the trigeminal ganglion itself. Within sensory ganglia the primary sensory neurons are tightly enveloped by a specialized cell, the satellite glial cell (SGC). In theory, if SGCs were induced to release GABA, then the activity of sensory neurons within the ganglion would be suppressed through activation of locally expressed GABA receptors. There are currently many attempts using cell-specific promoters to produce cell specific viral agents [9] but it is also known that some viruses have a 'natural' preference for a specific cell type [10-13]. We found an adenovector that preferentially infected SGCs and used this virus to transfer GAD65, one of the two GAD isoforms, into SGCs in order to produce GABA within the trigeminal ganglion.

We injected the adenovector directly into the trigeminal ganglion in order to infect SGCs then we confirmed the expression and location of GAD65 and GABA. To test the effects of the gene transfer on nociception, we used the orofacial formalin test, a standard model of inflammatory pain. To determine if the behavioral effect of the transgene occurred because of its expression within the trigeminal ganglion, we tested the effects of GABAA and GABAB receptor antagonists injected directly into the ganglion.

## Results

### Adenovirus-mediated expression of GAD65 occurs mainly in satellite glial cells (SGCs) in the trigeminal ganglion

**Figure 1:**
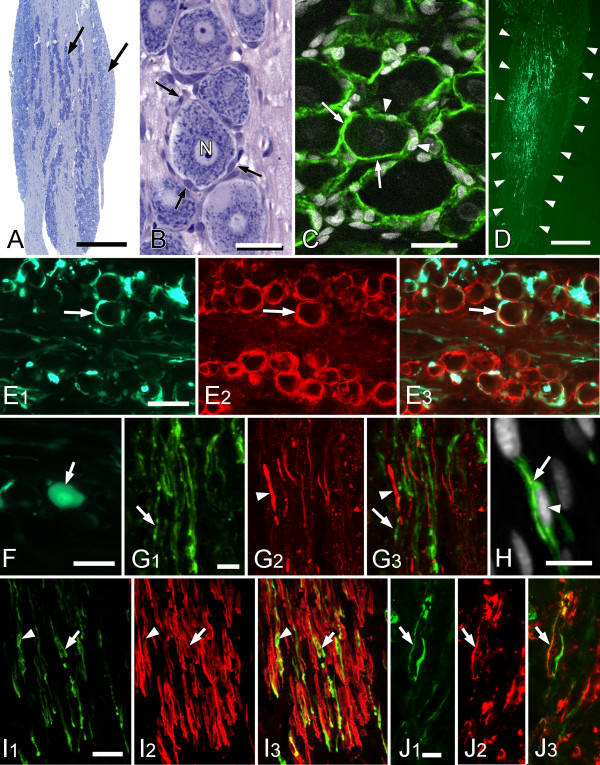
**Adenovirus-mediated expression of GAD65 in the trigeminal ganglion**. (**A**) Nissl stain showing the anatomical organization of the rat trigeminal ganglion with elongated clusters of sensory neurons (arrows) lying between bundles of myelinated axons. (**B**) At high magnification only the nuclei of SGCs (arrows) that surround the unipolar sensory neuron (N) are visible. (**C**) The attenuated cytoplasm of the SGCs is visible using the glial-specific marker, glutamine synthetase (arrows, green). Arrowheads point to SGC nuclei. (**D**) A low magnification image of the whole ganglion (outlined by arrowheads) showing the extent of GAD65 immunopositive elements (green) following injection of adGAD65. Images **E1-F3 **are taken 6 days after injection of AdGAD65 into the trigeminal ganglion. (**E1-E3**) GAD 65 expression (**E1**, green) is found in SGCs identified by GLAST immunostaining (**E2**, red) and in other elongated profiles (arrows). **E3 **Combined image. Asterisk indicates the same unstained cell body of a sensory neuron. (**F**) An example of a GAD65 positive neuron cell body. (**G1-G3**) The GAD65 immunopositive elongated process (**G1**, green) are not double labelled with the neuronal marker NF160 (**G2 **red) indicating that they are not axons. **G3 **combined image. Arrows and arrowheads indicate the same element is each figure. **H**, High magnification shows that the GAD65 immunopositive elongated elements (arrow) are often associated with nuclei (arrowhead). Double labelling for P75 (**I1**-**I3**) and Sch2e (**J1**-**J3**), a Schwann cell marker, show some GAD65 immunolabeled profiles are double labelled with these markers identifying the elongated profiles as non-myelinating (P75 positive) or myelinating (Sch2e positive) Schwann cells. Arrows and arrowheads indicate the same elements in each figure. Scale bars: A = 1 mm; B, C = 20 m; D = 1 mm; E, F = 40 μm; G, H = 10 μm; I = 20 μm; J = 5 μm.

The trigeminal ganglion is characterized by elongated clusters of sensory neurons lying between bundles of myelinated axons (Figure 1A). The cell bodies of primary sensory neurons are surrounded by satellite glial cells (SGCs). In Nissl preparations the nuclei of SGCs are visible but the cytoplasm surrounding the neurons is attenuated and not readily visible (Figure 1B). To identify the SGCs we used glial specific markers involved in glutamate metabolism, glutamine synthetase (Figure 1C) and glutamate transporter GLAST (Figure 1E2). Six days after injection of AdGAD65 into the trigeminal ganglion a region approximately 750 μm × 2500 μm showed GAD65 structures that included 50–70% of the SGCs (Figures 1D; E1–E3). Only an occasional GAD65 immunopositive neuron was seen (Figure 1F) but other, elongated, fiber-like immunopositive elements were present throughout the ganglion. To see whether the immunolabeled elements were axons we double stained using the NF160 neurofilament antibody (Figure 1G1–G3) and found none of the elongated structures were double labelled. The lack of axonal staining confirms the observation that only an occasional neuron was infected. Many of the elongated profiles appeared cellular (Figure 1H) and many of these double labelled with the neurotrophin receptor, P75 (Figure 1I), or the Schwann cell marker, Sch2E (Figure 1J), indicating that they were non-myelinating or myelinating Schwann cells. In some regions of the injected area the number of labelled SGCs and Schwann cells were approximately equal which suggests that a large number of myelinated and non-myelinated were associated with GAD65 immunopositive Schwann cells.

Immunohistochemistry for common markers of immune response showed that there was an increase in resident (ED2 positive) and circulating macrophages (ED1 positive) in addition to an infiltration of lymphocytes (TCR positive). The same amount of and types of immune cells were also seen in both AdGFP and AdLacZ injected trigeminal ganglia. The rats showed no systemic immune response.

### GAD65 expression in the trigeminal ganglion produces analgesia in the orofacial formalin test

**Figure 2:**
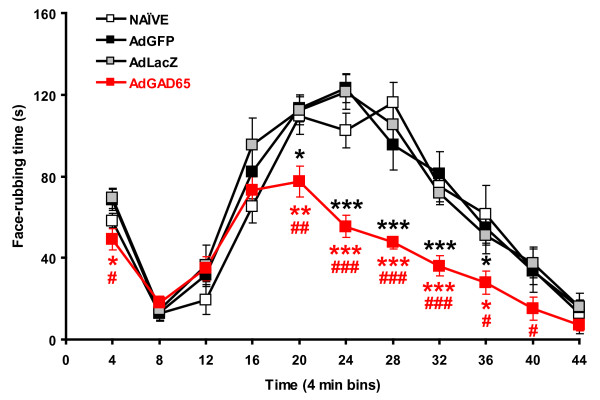
**Adenovirus-mediated expression of GAD65 in the trigeminal ganglion produces analgesia in the formalin test**. Orofacial formalin tests were performed six days after the injection of AdGAD65 or one of the control viruses (AdGFP or AdLacZ) and compared to Naïve rats. After injection of AdGAD65, there is a significant reduction of the nociceptive response (RM ANOVA: F = 3.8; P < 0.001) in the second phase of the orofacial formalin test. AdGAD65 vs. Naïve: **P < 0.01, ***P < 0.001; AdGAD65 vs AdGFP: *P < 0.05, **P < 0.01, ***P < 0.001; AdGAD65 vs AdLacZ: ##P < 0.01, ###P < 0.001.

The pain behavior induced by injection of formalin into the upper lip is characterized by two phases of intense pain behavior separated by an interphase of reduced pain behavior (Figure 2) [14]. The pain behavior consisted of face-rubbing (sustained face strokes of small amplitude) directed to the upper lip and whisker pad with the ipsilateral forepaw, often accompanied by the contralateral forepaw. Usually, the first phase is short-lasting (first 4 min of the test), while the second phase lasts for about 30 min starting 12 min after the beginning of the test.

**Figure 3:**
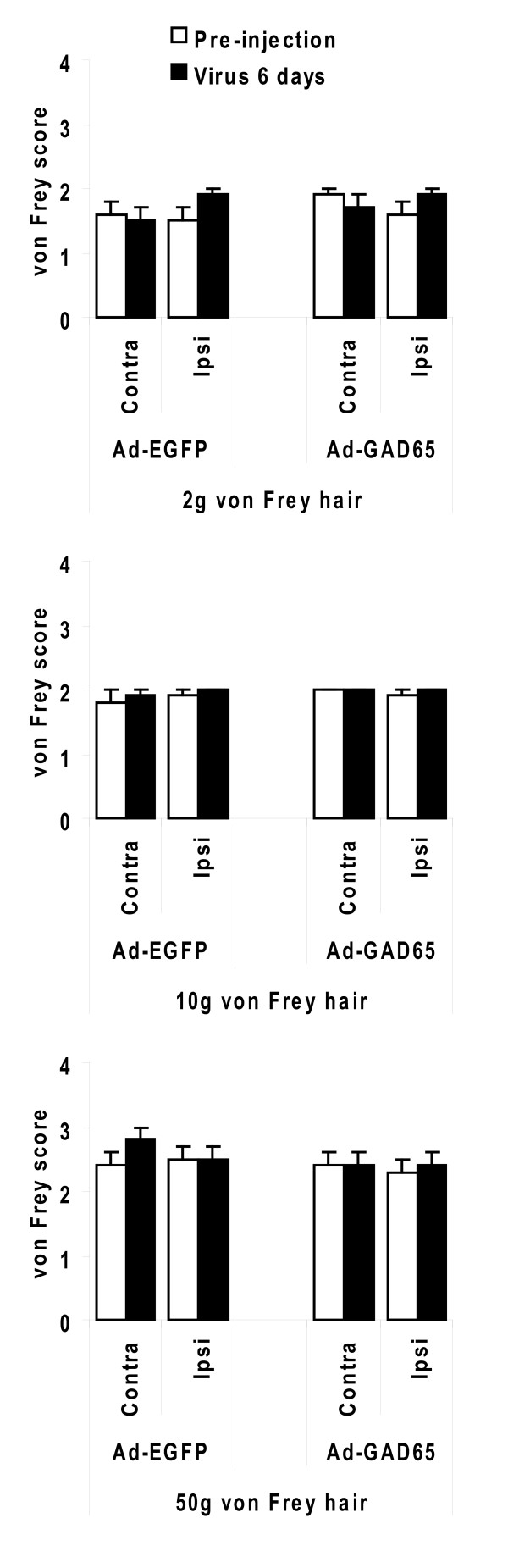
**Mechanical sensation of the face is unchanged after adenovirus-mediated expression of GAD65 in the trigeminal ganglion**. Six days after the injection of AdGAD65 or AdGFP, the rats were tested for their response to von Frey hair stimulation. For each hair, there was no difference in von Frey score between AdGAD65 and AdGFP when compared pre- and post-injection on the ipsilateral side (RM ANOVA: 2 g hair, F = 0.1, P = 0.791; 10 g hair, F = 0.0, P = 0.926; 50 g hair, F = 0.3, P = 0.566). Similarly, there was no difference between contralateral and ipsilateral von Frey score pre- and post-injection after either injection of AdGAD65 (RM ANOVA: 2 g hair, F = 2.1, P = 0.200; 10 g hair, F = 1.0, P = 0.356; 50 g hair, F = 1.0, P = 0.356) or AdGFP (RM ANOVA: 2 g hair, F = 2.3, P = 0.170; 10 g hair, F = 0.0, P = 1.000; 50 g hair, F = 4.2, P = 0.080).

When the formalin test was administered six days after the injection of AdGAD65 in the trigeminal ganglion, there was a significant decrease in the time spent face-rubbing during the second phase of the formalin test (from 12 to 36 min) when compared to naïve rats that received only a formalin injection (Figure 2). The injection of either control viruses (AdGFP or AdLacZ) did not affect any of the formalin phases and the pain behavior was comparable to that of naïve rats (Figure 2). The effect of injecting AdGAD65 into the trigeminal ganglion on the formalin test could not exclude the possibility that the effect was due to a loss of facial sensation rather than analgesia. We therefore carried out von Frey hair testing in adenovirus-injected rats (Figure 3) because in models of neuropathic pain, von Frey hair testing reveals allodynia caused by mechanical stimulation. We would predict that if loss of facial sensation occurred after adenovirus injection, there would be increase of the threshold to mechanical stimulation with von Frey hairs. Six days after injection of either AdGAD65 or AdGFP, three different hairs of increased stiffness were applied to the vibrissal pad of the rats. For each hair, there were no changes in the response of the rats to mechanical stimulation (Figure 3), confirming that the effect of AdGAD65 in the formalin test was an analgesic effect.

### GABAA and GABAB receptors are expressed in sensory neurons of the trigeminal ganglion

**Figure 4:**
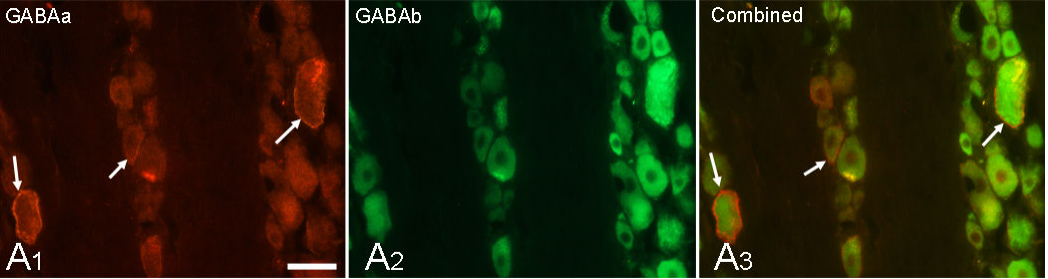
**Sensory neurons of the trigeminal ganglion express both GABAA and GABAB receptors**. GABAA receptor immunostaining of ganglion neurons is membrane associated (**A1**, red, arrows) while GABAB immunostaining is cytoplasmic **(A2) **The combined image **(A3) **shows some neurons are double labelled for GABAA receptor and GABAB receptor (**A3**, arrows). Scale bar = 30 μm

In view of the previous results, we sought to determine if AdGAD65 produced its antinociceptive effect locally in the trigeminal ganglion through the activation of GABA receptors. We first confirmed that GABAA and GABAB receptors were expressed in sensory neurons of the trigeminal ganglion (Figure 4). GABAA receptor expression appeared as light cytoplasmic immunostaining with stronger intensity at the surface of the cell bodies of the sensory neurons (Figure 4A and 4C). The expression of GABAB receptors was only found in the cytoplasm of sensory neurons (Figure 4B and 4C). Both GABAA and GABAB receptors were seen in all types of sensory neurons.

### GAD65-mediated analgesia is reversed by injection of bicuculline, a GABAA receptor antagonist

**Figure 5:**
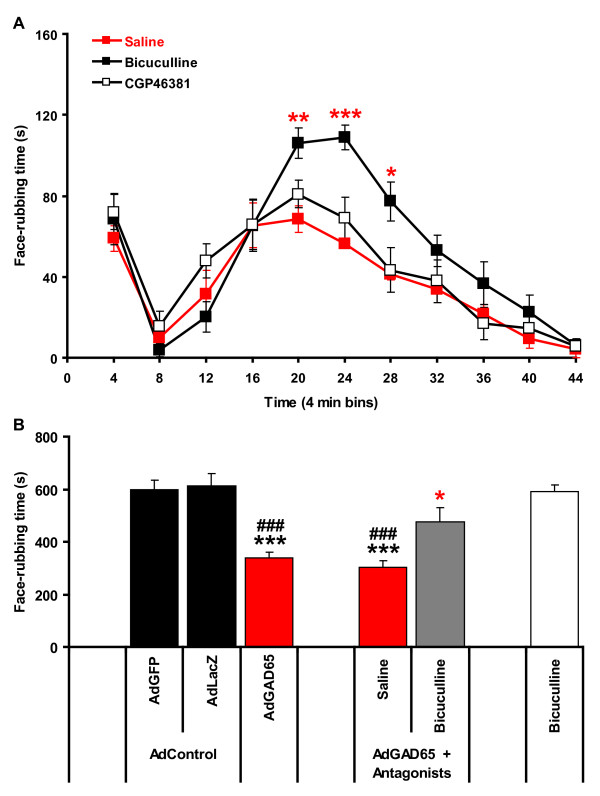
**GAD65-mediated analgesia is reversed by the GABAA receptor antagonist bicuculline**. (A) Six days after injection of AdGAD65 into the trigeminal ganglion, a second injection was performed with saline, bicuculline or CGP46381 (a GABAB receptor antagonist), and the orofacial formalin test was carried out 10 minutes later. Bicuculline, but not CGP46381 reversed the AdGAD65-mediated analgesia in the second phase of the formalin test (RM ANOVA: F = 2.7; P < 0.001). AdGAD65 + bicuculline vs AdGAD65 + saline: **P < 0.01, ***P < 0.001. (**B**) Comparison of the averaged pain behavior in the second phase of the formalin test shows a significant difference between groups (one-way ANOVA: F = 15.6; P < 0.001). AdGAD65 produced a significany decrease in pain behavior. Bicuculline reverses AdGAD65-mediated analgesia. Bicuculline alone does not affect pain behavior. AdGAD65 or AdGAD65 + saline vs. AdGFP: ***P < 0.001; AdGAD65 or AdGAD65 + saline vs AdGFP: ###P < 0.001; AdGAD65 + saline vs. AdGAD65 + bicuculline: *P < 0.05.

The finding that adenovirus-mediated expression of GAD65 occurred principally in SGCs together with the presence of GABA receptor in the cell bodies of sensory neurons suggested that GABA might produce its antinociceptive effect inside the trigeminal ganglion in a paracrine manner. To test this possibility, we injected rats with AdGAD65, and 6 days later made a second injection into the trigeminal ganglion with either saline, bicuculline (a GABAA receptor antagonist) or CGP46381 (a GABAB receptor antagonist) followed 10 minutes later by the orofacial formalin test. When bicuculline (200 pmol in 2 μl) was injected into the trigeminal ganglion, GAD65-produced analgesia was reversed in the formalin test (Figure 5). The pain behavior observed from 12 to 28 min post-injection of formalin was higher after injection of bicuculline when compared to injection of saline (Figure 5A). On the contrary, the injection of CGP46381 (10 nmol in 2 μl) into the trigeminal ganglion had no effect on GAD65-mediated analgesia in the orofacial formalin test (Figure 5A). The reversion of GAD65-mediated analgesia by bicuculline was maximal as shown by the total pain behavior in the second phase of the formalin test (Figure 5B). In rats receiving AdGAD65 and bicuculline, the total pain behavior in the second phase of the formalin test (from 12 to 44 min) was similar to that from rats injected with either AdGFP or AdLacZ control adenoviruses (Figure 5B). Bicuculline alone in normal rats had no effect on the formalin test and the pain behavior in the second phase of the test was identical to that of control animals (Figure 5B).

## Discussion

The present study shows that adenoviral transfer of the GAD65 gene into satellite glial cells (SGCs) of the trigeminal ganglion is sufficient to reduce acute pain behavior resulting from the orofacial formalin test. The application of GAD viral vectors has previously been used to produce analgesia but these studies differ from the present study in two key aspects. Firstly, in the current study the adenoviral transfer occurs principally in SGCs rather than neurons and secondly, the antinociceptive effect is mediated at the level of the ganglion via GABAA receptors.

### Viral Targeting

The adenoviral vector used here was not specifically designed to target glial cells, but following trigeminal ganglion injection many SGCs expressed GAD65 while very few transfected neurons were seen. Other studies using adenoviral injection into the central nervous system have reported a similar result [15,16] while others have reported transfection limited to neurons [17] or to both neurons and glia [18]. It has been pointed out that factors affecting adenoviral target-cell specificity has not been fully explored and that some viruses have a 'natural' preference for a specific cell type [10-12]. Part of the cell specificity in our study might result from the fact that direct injection into the ganglion makes the virus available to all cell types whereas peripheral injection specifically targets neurons via the peripheral branches of their axons. In addition, because the SGCs tightly envelop neurons (see below) they might present a physical barrier preventing access to the neurons. Whatever the explanation for the SGC specific adenoviral uptake, the idea that glial cell transfection alone can cause behavioural changes in absence of neuronal transfection, opens up further possibilities for virally mediated therapeutics.

### The SGC and neurons

The classic notion that glial cells serve principally as support cell for neurons has changed over the last few decades with evidence of the many roles glial cells play in maintaining proper neuronal function [19-21]. In the sensory ganglia, SGCs and the neurons they surround are so intimately associated they have been described as a functional unit with the SGCs playing a key role in modulating the perineuronal environment [22,23]. This close relationship makes SGCs an obvious target for the treatment of different pain conditions at is reasonable to suppose that GABA produced by SGCs will have immediate access to the neurons they envelop that are separated from the SGCs by an extracellular space as small as 20 nm. However for these steps to occur the SGCs must have the necessary components to synthesize GABA and GABA receptors must be present on the neurons. In fact, like astrocytes in the CNS, SGCs participate in glutamate metabolism through the presence of glutamine synthetase and take up glutamate through a glial-specific glutamate transporter, GLAST [23,24]. In normal conditions, the glutamate is converted to glutamine by glutamine synthetase (Figure 1 and [22]) but with the additional presence of GAD, the SGCs have the necessary components to produce GABA. *In vitro *studies confirm that astrocytes infected with either GAD-expressing HSV or HFV retrovirus are able to synthesize GABA from glutamate [25-27]. The ability of SGCs to release GABA was concluded after exogenously administered ^3^H-GABA was shown to be released in the vicinity of sensory neurons [28-30]. In this latter case, the secretion of GABA in the perineuronal environment was dependent on neuronal excitation and occurred with the increase in extracellular potassium concentrations.

### The sensory neuron and local GABAergic systems

Primary neurons in sensory ganglion are known to possess both GABAA and GABAB receptors [31-35] but there is some question over whether neurons or glia in the ganglia normally produce GABA. Although there are several reports that GAD65 and GABA are present in both neurons and SGCs of sensory ganglia [4,36,37], these papers have not been fully substantiated by other studies. For example, in contrast to the above studies, Liu and colleagues [6,8,38] found GAD immunostaining of the ganglia was not above background but the same group did detect low levels of GAD by real time PCR and Western blotting and also found low level of GABA release into the dorsal horn [6,8,38]. There is also a consensus from studies of the spinal dorsal horn that primary afferents are not GABA or GAD immunopositive [39-41]. Our own results showing that immunostaining for GAD65 is not above background in control trigeminal ganglia also supports the idea that GAD is, at best, expressed at very low levels in the ganglia and the functional significance is unclear.

### Location of action, ganglia or spinal cord?

Several previous studies have shown that introducing GAD genes into sensory neuron is effective in reducing pain behavior of peripheral and central origin [6-8,25,38,42]. In these cases, the GAD containing viral vector was delivered to peripheral targets, transported to primary sensory neuronal cell bodies and the resulting analgesia was determined to result from the release of GABA from primary afferent into the dorsal horn of the spinal cord. We believe that in the present study the antinociceptive effect is mediated by intraganglionic processes. The principal evidence is that GAD65 expression was present principally in SGCs and only occasionally in neurons. Second, the reversal of the AdGAD65-mediated antinociception by bicuculline (a potent GABAA receptor antagonist) injected directly into the trigeminal ganglion also points to a local, ganglionic site of action. Direct ganglion injection of CGP46381, a GABAB antagonist, had no effect showing that the analgesia is mediated by GABAA receptors.

The idea that altering GABA within sensory ganglia can produce analgesia is relatively new, but recently Naik and colleagues [3] showed that direct injection of a GABAA receptor agonists into the DRG was antinociceptive in a model of peripheral nerve injury. The antinociceptive effect of the GABAA receptor agonists was blocked by the intraganglionic injection of the competitive GABAA receptor antagonist, bicuculline. Naik et al [3] also showed that muscimol given at the time of injury resulted in a long lasting reduction of neuropathic pain while muscimol given after the development of neuropathic pain only resulted in a short-lived reduction in pain. Our results confirm this latter finding of Naik and colleagues showing that GABA or GABA agonists applied directly to the ganglion can produce analgesia, not only for nerve injury induced neuropathic pain but also for inflammatory pain.

### Mechanism of antinociceptive action

The most straightforward explanation for the AdGAD65-mediated analgesia is that GABA released by SGCs inhibits tonic and/or induced neuronal firing of nearby neurons and thus reduces nociceptive activity. However, the most profound antinociceptive effect is in the second phase of the formalin test and if the AdGAD65 effect was to reduce all neuronal activity one would expect to also see an effect in the first phase. The second phase of the formalin test is believed to represent tissue damage-induced spinal sensitization, resulting in facilitated pain processing [2]. Activation of nociceptive primary neurons by subcutaneous injection of formalin leads to central release of excitatory neurotransmitters or neuromediators such as glutamate, substance P and ATP responsible for the induction and maintenance of central sensitization [1,43]. In addition to the central release of neurotransmitters, it has been shown that glutamate, substance P and ATP can be released inside sensory ganglia following activation of primary sensory neurons [44-50]. Many of these released substances are excitatory, for example, it has been suggested that substance P could act in a paracrine manner in sensory ganglia to activate neurokinin-1 receptors on nociceptive neurons [51]. Because the effect of GABA on primary sensory neurons is generally depolarising it is not yet clear how an analgesic effect would be achieved. Naik and colleagues have suggested that the GABA mediated depolarization might clamp the membrane at the reversal potential of Cl^- ^and thus inactivate voltage sensitive channels such Na^+ ^and Ca^2+ ^[3]. An alternative proposal offered by Naik and colleagues, that the GABA-activated Cl^- ^currents inhibit the excitatory effects of ATP[3], is supported by our findings that the strongest analgesic effect is on the second, ATP related, phase of the formalin test. Whatever the mechanism, it is clear that in our experimental conditions the effect of GABA in the ganglion preferentially targets nociceptive neurons, because the effect is analgesic rather than anaesthetic. It might be that the continued presence of GABA in the ganglion dampens, rather than completely abolishes neuronal activity and reduces ongoing activity resulting from nerve damage that eventually leads to peripheral or central sensitization.

## Conclusion

The use of non-pharmacological approaches to treat disease, including pain, is only just beginning to be explored. Such approaches hold great promise in their target specificity both in terms of the molecules and cell types. The current study shows the efficacy of such an approach in an animal model and also highlights the important role that SGCs, traditionally regarded as support cells, might play in a therapeutic strategy.

## Methods

### Sub- heading for this section

#### 4.1 Animals

Adult male Sprague-Dawley rats (Charles River) weighing between 300 and 350 g were housed on a 12 h light/dark cycle and given food and water *ad libitum*. Procedures followed the NIH Guidelines for the Care and Use of Laboratory Animals and were approved by the University of California, San Francisco and Cedars-Sinai Medical Center Institutional Animal Care and Use Committee.

#### 4.2 Cannula guide implantation

Rats were anesthetized with a mixture of ketamine (75 mg/kg) and medetomidine (0.5 mg/kg) injected intraperitoneally. The head was placed in a stereotaxic frame, and a 2 cm-long midline incision was made in the skin. Using stereotaxic guidance, a burr hole (approximately 2 mm in diameter) was drilled above the location of the maxillary division of the left trigeminal ganglion at 6.5 mm anterior to interaural 0 and 2.3 mm from the midline. A guide cannula was then anchored onto the skull using three stainless steel screws and dental cement. At least 5 days were allowed for recovery from surgery before injection into the trigeminal ganglion.

#### 4.3 Adenoviral vectors

We used a recombinant adenovirus encoding the human 65 kDa isoform of GAD (GAD65) under the transcriptional control of chicken actin ubiquitous promoter (AdGAD65) as generated previously [52]. Two recombinant adenovirus, expressing either green fluorescent protein (AdGFP) or β-galactosidase (AdLacZ) under the control of cytomegalovirus (CMV) promoter were used as controls. AdGFP and AdLacZ were produced at Gene Therapeutics Research Institute. The backbone of all three viruses was adenoviruses type 5 with deletions in E1 and E3. All adenoviruses were grown and purified as previously described ([53]). Rats were injected into the left trigeminal ganglion (see below) with 5 × 10^7 ^pfu of adenovector.

#### 4.4 Drugs

The selective GABAA receptor antagonist, (-) bicuculline methiodide , and the selective GABAB receptor antagonist, CGP46381 (3-amino-propyl-(cyclohexylmethyl)-phosphonic acid; ), were diluted in sterile saline (0.9% NaCl) at a concentration of 100 μM (50 μg/ml) and 5 mM (1.2 mg/ml), respectively. A volume of 2 μl was administered into the trigeminal ganglion of awake animals (as described below) corresponding to 200 pmol (100 ng) of bicuculline or 10 nmol (2.4 μg) of CGP46381.

#### 4.5 Trigeminal ganglion inoculation

Injections into the trigeminal ganglion were done with the rats either under gas anesthesia or awake. For injection of adenovirus, rats were lightly anesthetized with an inhalant anesthetic, delivered through a face mask (1–2% isoflurane-40% oxygen). For injection of GABA receptor antagonists, awake rats were gently restrained in a transparent, cone-shaped plastic bag with a tube attached to the tip of the cone delivering oxygen at a rate of 4 L/min. The cone had an opening so that the guide cannula was accessible. In all cases, a 33-gauge beveled stainless steel internal cannula was inserted into the guide cannula (positioned over the left trigeminal ganglion as described above) to 10.5 mm below the cortical surface. The internal cannula was connected to a 25 μl syringe driven by a microinjection pump set to deliver 2 μl over a 1 min period. Rats were maintained with food and water ad libitum for 6 days after injection of adenovirus.

#### 4.6 Orofacial formalin test

We followed a protocol modified from Clavelou et al. [14]. One day prior to the formalin test, the rats were acclimated for 1 h to the testing chambers (44 × 24 × 24 cm). On the testing day, 50 μl of 2.5% formalin solution in saline was injected subcutaneously with a 30-gauge hypodermic needle into the left upper lip (side of intraganglionic injection of virus), lateral to the midline. The spread of the formalin solution has been previously described [54]. The animal was then immediately placed into the testing chamber and continuously observed for 44 min, during which time the nociceptive behavior (i.e. face-rubs) was quantified. Data were collected using a computer program that automatically records the pain behavior in successive 4 min bins. One hour after formalin injection the rats were euthanized and perfused for histological examination of the trigeminal ganglia.

#### 4.7 Mechanical (von Frey hair) stimulation

Rats were tested for response to von Frey hair application as described previously [55]. Briefly, three von Frey hairs of increasing stiffness, 2, 10 and 50 g, were applied five consecutive times on different areas of the vibrissal pad and in the perioral and perinasal territory. The behavioral response of the rats was scored according to Vos et al. (1994). For each hair, the highest score was recorded and the results for each hair are presented separately.

#### 4.8 Statistics

All data are expressed as mean ± SEM and were analyzed using SigmaStat software (Systat Software, San Jose, CA). In the formalin test, differences between groups over time were analyzed using a mixed repeated-measure (RM) analysis of variance (ANOVA) with Time as the repetition factor. When significant, the RM ANOVA was followed by multiple comparisons between groups using Bonferroni's *post hoc *tests. For the analysis of the 2^nd ^phase of the formalin test, a one-way ANOVA was performed to assess the difference among groups, followed by Bonferroni's *post hoc *tests for multiple comparisons between groups. Results were considered statistically significant at P < 0.05. For von Frey hair testing, differences between the two adenovirus groups (EGFP and GAD65) and changes over time were analyzed for each hair by mixed RM ANOVA, followed by Bonferroni's post hoc comparisons between groups. In each group, differences between ipsilateral and contralateral sides to the injection were tested over time with a two-way RM ANOVA.

#### 4.8 Perfusion and fixation

The rat was euthanized by deep anesthesia with an intraperitoneal injection of pentobarbital (100 mg/kg) followed by perfusion transcardially with Tyrode's solution followed by 10% formalin. The left and right trigeminal ganglia were postfixed in the same fixative for 30 min, and then transferred in a solution of 30% sucrose in PBS, pH 7.4 for 48 hours.

#### 4.9 Tissue processing and immunohistochemistry

After cryoprotection in sucrose, the left and right trigeminal ganglia were frozen and embedded together in OCT compound. The right trigeminal ganglion was used as internal control for protein expression. The block containing both ganglia was cut at a thickness of 10 μm on a cryostat. The sections were collected on slides. For Nissl staining section were stained for 5 minutes in 2% Cresyl Violet then dehydrated and coverslipped using standard protocols. For immunohistochemistry, on-slide sections were blocked for 1 hour in 5% normal goat serum (NGS) and 0.3% Triton X-100 in PBS, pH 7.4, and then incubated overnight in the primary antiserum: GAD65 (1:1,000), glutamine synthetase (1:5,000; AbCam), P75 (1:1000, Chemicon), Schwann2E (1:1000 Developmental Studies Hybridoma Bank, Baltimore), GLAST (1:16,000; Millipore), neurofilament 160 (NF160) (1:200; Sigma), GABAA receptor (1:500; Chemicon) and GABAB receptor ((1:10,000, Dr. Margeta-Mitrovic[56]) in 5% NGS and 0.3% Triton X-100. For immunofluorescence, a FITC- or Cy3-conjugated secondary antibody (Jackson ImmunoResearch), diluted 1:400 in 5% NGS and 0.3% Triton/PBS, was used for 1 h. For amplification, a biotinylated secondary antibody, diluted 1:400 in 5% NGS and 0.3% Triton/PBS, was used for 1 hour, then the sections were washed and incubated for 1 hour in a 1:400 ABC Elite solution diluted in 0.3% Triton/PBS. Amplification was performed by placing sections in biotinylated tyramide for 5 min, followed by incubation for 1 hour in FITC-streptavidin solution, diluted 1:400 in PBS. Sections were then washed and coverslipped with Vectashield. Omitting the primary antibody controlled for non-specific labeling.

## Competing interests

The authors declare that they have no competing interests.

## Authors' contributions

J-PV, PTO, CS and LJ participated equally in the experimental design, performing experiments, and analysis and writing the manuscript. BR, PM, CL, MP, PL and MC designed and produced the viral vectors used in the study.
